# Left Atrial Mass Invasion from Pulmonary Neoplasm Extension via the Right Upper Pulmonary Vein Presenting as Ipsilateral Stroke

**DOI:** 10.1155/2016/7084234

**Published:** 2016-12-08

**Authors:** Piercarlo Ballo, Raffaele Laureano, Mariapia Briganti, Maria Teresa Passaleva, Fiorella Piani, Cecilia Piga, Stefano Tatini, Giovanni Maria Santoro

**Affiliations:** ^1^Cardiology Unit, S. Maria Annunziata Hospital, Florence, Italy; ^2^Department of Medicine, S. Maria Annunziata Hospital, Florence, Italy

## Abstract

Left atrial invasion by lung cancer via haematogenous pathways is a relatively uncommon but potentially life-threatening event. While several cardiac complications of cardiac involvement have been previously described, the evolution towards cerebral stroke has been rarely reported. In this case report, we describe an atypical case of haematogenous metastatic invasion of the left atrium from pulmonary neoplasm extension presenting as an ipsilateral stroke whose ASCO classification changed during the clinical management.

## 1. Introduction

Although cardiac metastases have been reported in up to 25% of patients with lung cancer in autoptic studies [1], the detection of cardiac involvement in these patients is relatively uncommon in clinical practice [2–4]. The metastatic pathway to the heart is often lymphatic, but hematogenous patterns can also be observed [5]. From a clinical point of view, invasion of the left heart may be a life-threatening event, potentially leading to a number of complications such as obstructed pulmonary venous flow [6], cardiac tamponade [7], ventricular arrhythmias [8], complete atrioventricular block [9], left ventricular inflow obstruction [10], and myocardial infarction [11].

Cerebral stroke as a result of systemic embolization from the left heart has been exceptionally reported [12] and may sometimes represent the first clinical presentation of the neoplasm. Adequate identification of the underlying cause of stroke is therefore of major clinical importance in these cases. Compared with the old Trial of ORG 10172 in Acute Stroke Treatment (TOAST) classification system, newer classification schemes such as the ASCO (A, atherosclerosis; S, small vessel disease; C, cardiac source; O, other causes) phenotypic system can facilitate the identification of the most likely cause by grading the probability of each factor and accounting for the extent of the diagnostic work-up [13]. This approach may be particularly useful when multiple potential mechanisms are present, since it reduces the prevalence of patients with stroke of indeterminate origin. In this report, we describe an unusual case of left atrial (LA) invasion from pulmonary neoplasm extension via the right upper pulmonary vein whose first clinical presentation was characterized by an ipsilateral stroke with evolving ASCO categorization during the management.

## 2. Case Report

A 76-year-old man presented to the Emergency Department because of left hemiparesis and dysarthria. His history was relevant for past smoking, systemic hypertension, and laryngeal cancer treated by total laryngectomy and tracheostomy 5 years earlier. His usual therapy included aspirin, losartan, and doxazosin. During the last 3 weeks, he had shown recurrent episodes of postural instability and paresthesia in the left arm. A brain computed tomography (CT) performed 2 weeks earlier had shown no significant abnormalities. At the current examination, chest and cardiac examinations were normal, blood pressure was 170/75 mmHg, heart rate was 77 bpm, body temperature was 36.5°C, and oxygen saturation was 95%. The ECG was normal. Neurological examination showed facial-brachial-crural left hemiparesis (NIH Stroke Scale = 8). A new brain CT showed an ipsilateral hypodense lesion in the left semioval center. The patient was hospitalized, and therapy with aspirin, methylprednisolone, ramipril, and dalteparin was started. An echo-Doppler of supra-aortic vessels showed subcritical stenosis of the right internal carotid artery and critical stenosis of the left common carotid artery with occlusion of proximal left internal carotid artery. An atherosclerotic stroke was diagnosed (ASCO A1b).

**Figure 1:**
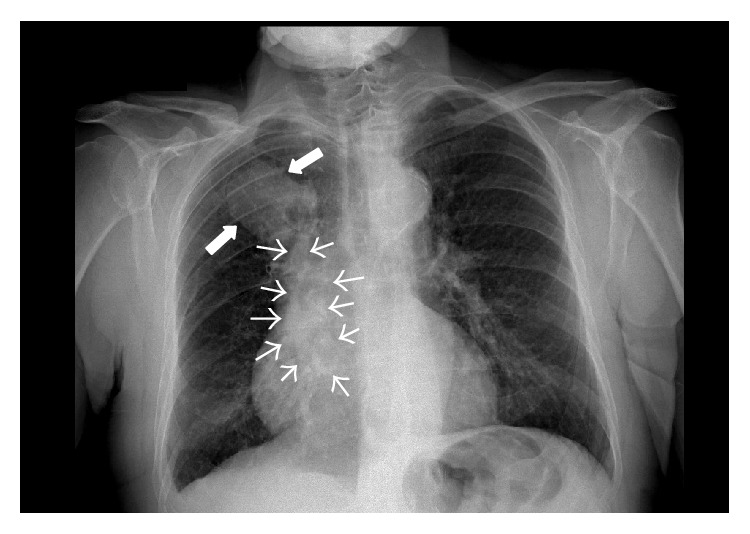
Chest X-ray showing a rounding mass projecting over the right upper lobe and extending towards the right upper portion of cardiac silhouette.

**Figure 2:**
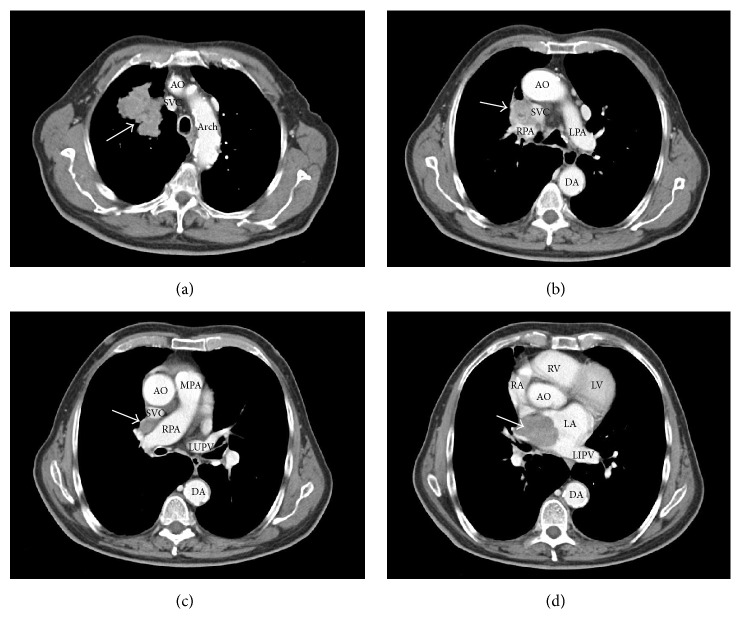
Chest CT showing a large opacity with irregular borders in the right upper lobe (a), with invasion of the right upper pulmonary vein (b-c) and extension into the left atrium (d). The arrows indicate the mass along its pathway from the right upper pulmonary lobe to the left atrium (LA). Note the lack of contrast signal in the right upper pulmonary vein, obstructed by the mass. AO, aorta; DA, descending aorta; LAA, left atrial appendage; LPA, left pulmonary artery; LIPV, left inferior pulmonary vein; LUPV, left upper pulmonary vein; LV, left ventricle; MPA, main pulmonary artery; RA, right atrium; RPA, right pulmonary artery; RV, right ventricle; SVC, superior vena cava.

**Figure 3:**
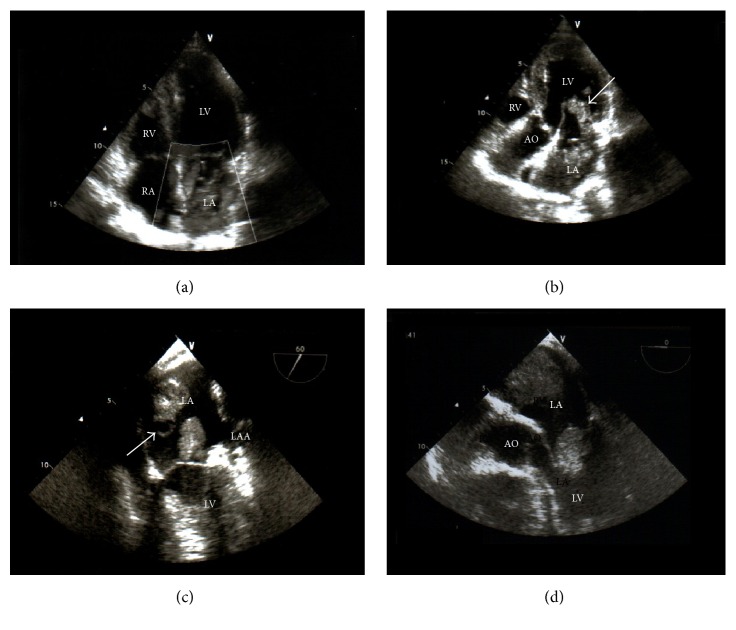
((a) and (b)) Transthoracic echocardiography showing massive left atrial invasion by a large multilobed mass, with protrusion through the mitral valve into the left ventricle during diastole (arrow). ((c) and (d)) Transesophageal echocardiography confirming left atrial invasion by a large mass entering the left ventricle in diastole and showing regions of cystic colliquation within the mass (arrow). AO, aorta; LA, left atrium; LAA, left atrial appendage; LV, left ventricle; RA, right atrium; RV, right ventricle.

**Figure 4:**
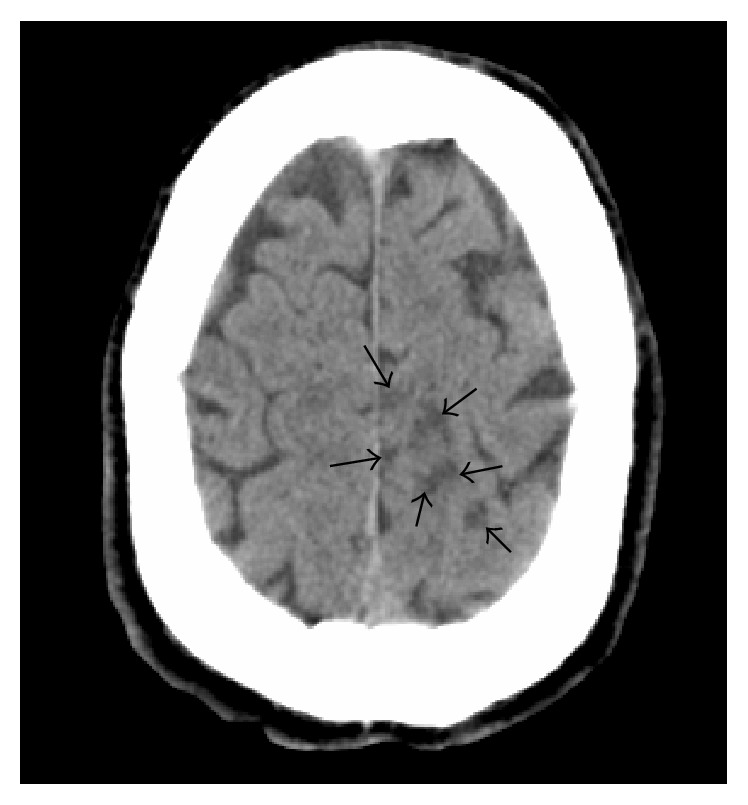
Brain CT showing new multiple, diffuse hypodense lesions near the vertex in the left hemisphere (arrows).

**Figure 5:**
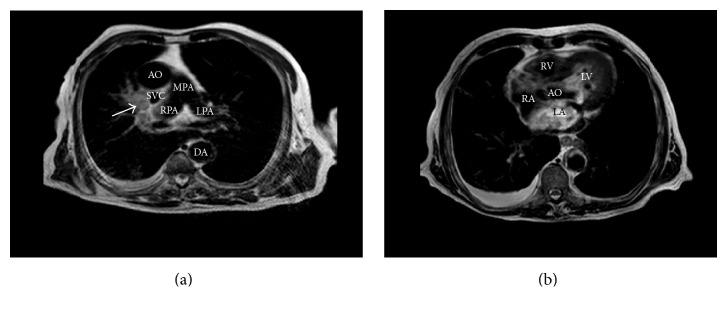
Chest magnetic resonance imaging showing invasion and subtotal obliteration of left atrial cavity from the right upper pulmonary vein (arrow). AO, aorta; DA, descending aorta; LA, left atrium; LPA, left pulmonary artery; LV, left ventricle; MPA, main pulmonary artery; RA, right atrium; RPA, right pulmonary artery; RV, right ventricle; SVC, superior vena cava.

During the following hours, neurological conditions progressively improved. Routine chest X-ray evidenced a rounding mass projecting over the right upper lobe and extending towards the right upper portion of cardiac silhouette (Figure 1). Chest CT confirmed the presence of a large opacity with irregular borders, with invasion of the right upper pulmonary vein and extension into the left atrium (Figure 2). Transthoracic echocardiography showed massive LA invasion by a large, multilobed, highly mobile mass (48 × 35 mm) protruding through the mitral valve into the left ventricle during diastole (Figures 3(a) and 3(b)). Transesophageal echocardiography confirmed LA invasion with occlusion of the right upper pulmonary vein and pericardial infiltration through the LA roof, allowing visualization of areas of vascularization and some regions of cystic colliquation within the mass (Figures 3(c) and 3(d)). The stroke was reclassified as ASCO C_1_. On day 7, sudden right hemiparesis with spatiotemporal disorientation occurred. Brain CT showed new multiple, diffuse hypodense lesions near the vertex in the left hemisphere (Figure 4). After careful clinical evaluation, the patient was considered at too high risk to undergo thoracic surgery. On day 20, cardiac magnetic resonance showed a further increase in the dimension of LA mass with subtotal obliteration of LA cavity (Figure 5). On day 23, the patient died because of cardiac arrest.

## 3. Discussion

Although relatively rare, metastatization of pulmonary neoplasm to the left atrium has been well documented, particularly in patients with primary lung cancer [14, 15]. In a previous review of 215 lung cancer patients studied by gadolinium-enhanced 3D magnetic resonance angiography, an involvement of the proximal portion of the pulmonary veins and an extension into the left atrium were found in 9 (4.2%) and 2 (0.9%) patients, respectively [16]. Similarly, a more recent retrospective analysis of 4668 patients who underwent surgery for lung cancer found pathological evidence of pulmonary vein and LA involvement in 34 (0.7%) and 25 (0.5%) subjects, respectively [17]. LA invasion usually occurs by two main mechanisms, including direct infiltration of myocardial tissue by contiguity [18–20] and extension into the left atrium via the lymphatics and/or the pulmonary veins [21–28]. Patients most commonly suffer symptoms related to lung cancer (e.g., cough, hemoptysis, and weight loss) or sometimes related to cardiac complications as the first clinical presentation. A limited number of reports previously described cardiovascular presentations secondary to systemic neoplastic embolization, including cerebral ischemia [29–31] or peripheral arterial occlusion [32]. Noteworthy, most previous reports of cerebral ischemia described events with typical contralateral presentation [12, 29] or incidental detection of brain ischemic lesion by imaging techniques in asymptomatic subjects [30, 31]. Although patients with metastatic involvement of the heart generally have poor clinical outcome, their management should include a careful assessment of surgical options. When appropriate, the treatment of choice is complete resection in combination with chemotherapy or radiotherapy [32]. However, in the majority of cases, cardiac metastases occur in patients with advanced neoplastic disease who have already undergone resection of the tumor of origin. In these cases, cardiac treatment is usually confined to palliative interventions to relieve cardiac compression or haemodynamic obstruction if indicated. Moreover, complete resection of the tumor is not always possible, and postoperative mortality is relatively high [33, 34].

In this report, we describe the case of a primary lung neoplasm extending into the left atrium via a pulmonary vein and complicated by stroke presenting as ipsilateral hemiparesis. Several atypical issues should be pointed out in this case: (1) the clinical presentation as stroke with left hemiparesis and CT evidence of ipsilateral acute ischemic lesion, which might suggest the presence of uncrossed corticospinal tracts in our patient [35]; (2) the changes in stroke categorization according to the ASCO classification, related to the detection of left internal carotid artery occlusion with successive evidence of cardiac source of cerebral embolization; (3) the successive clinical evolution with sudden-onset right hemiparesis associated with multiple contralateral left-sided lesions, suggestive for an embolization pattern; (4) the growth rate of LA mass, which rapidly led to LA cavity obliteration and cardiac death. It should be pointed out that since tissue analysis data were not available, caution is needed in interpreting these findings. Although both the clinical course and imaging data support the hypothesis of a metastatic nature of the mass, without histologic confirmation, the diagnosis cannot be considered as definitively established. From a practical point of view, this report highlights the importance of considering the ASCO classification as a dynamic tool to define the phenotypic nature of stroke and of considering echocardiography as a cornerstone in the evaluation, diagnosis, and management of patients with clinical evidence of cerebral ischemia [36].
